# The Antiosteoporosis Effects of Yishen Bugu Ye Based on Its Regulation on the Differentiation of Osteoblast and Osteoclast

**DOI:** 10.1155/2020/9467683

**Published:** 2020-02-19

**Authors:** Yangyang Li, Yongfeng Zhang, Weiqi Meng, Yutong Li, Tao Huang, Di Wang, Min Hu

**Affiliations:** ^1^Department of Orthodontics, School and Hospital of Stomatology, Jilin University, Changchun 130021, China; ^2^School of Life Sciences, Jilin University, Changchun 130012, China; ^3^Changchun University of Chinese Medicine, Changchun 130117, China

## Abstract

Yishen Bugu Ye (YSBGY), a traditional Chinese medicine comprising 12 types of medicinal herbs, is often prescribed in China to increase bone strength. In this study, the antiosteoporotic effects of YSBGY were investigated in C57BL/6 mice afflicted with dexamethasone- (Dex-) induced osteoporosis (OP). The results showed that YSBGY reduced the interstitial edema in the liver and kidney of mice with Dex-induced OP. It also increased the number of trabecular bone elements and chondrocytes in the femur, promoted cortical bone thickness and trabecular bone density, and modulated the OP-related indexes in the femur and tibia of OP mice. It also increased the serum concentrations of type I collagen, osteocalcin, osteopontin, bone morphogenetic protein-2, bone morphogenetic protein receptor type 2, C-terminal telopeptide of type I collagen, and runt-related transcription factor-2 and reduced those of tartrate-resistant acid phosphatase 5 and nuclear factor of activated T cells in these mice, suggesting that it improved osteoblast differentiation and suppressed osteoclast differentiation. The anti-inflammatory effect of YSBGY was confirmed by the increase in the serum concentrations of interleukin- (IL-) 33 and the decrease in concentrations of IL-1, IL-7, and tumor necrosis factor-*α* in OP mice. Furthermore, YSBGY enhanced the serum concentrations of superoxide dismutase and catalase in these mice, indicating that it also exerted antioxidative effects. This is the first study to confirm the antiosteoporotic effects of YSBGY in mice with Dex-induced OP, and it showed that these effects may be related to the YSBGY-induced modulation of the osteoblast/osteoclast balance and serum concentrations of inflammatory factors. These results provide experimental evidence supporting the use of YSBGY for supporting bone formation in the clinical setting.

## 1. Introduction

Osteoporosis (OP) is a metabolic skeletal disorder characterized by decreased bone mass and microstructural destruction of bone tissue [1] and can be divided into primary and secondary OP [2]. Although OP can occur in people of all ages, it is mainly found in postmenopausal women and elderly men [3]. OP affects approximately 75 million people in the United States, Europe, and Japan [4]. Together with the increasingly aging population of China, this means that approximately 100 million people are estimated to have low bone mass or OP [5]. To date, the burdens of OP and its associated fracture-related morbidity and mortality have become a serious socioeconomic problem worldwide.

Osteoblasts and osteoclasts work synergistically to maintain bone homeostasis. Increase in osteoclast-mediated bone resorption and decrease in osteoblast-mediated bone formation disrupt the osteoclast/osteoblast balance, leading to OP [6, 7]. During the bone formation process, osteoblasts form new bone directly through the synthesis and secretion of bone-associated proteins and also indirectly control osteoclast-mediated bone resorption [7]. Oxidative stress also regulates bone homeostasis, and a redox imbalance can promote osteoblast apoptosis and induce osteoclast differentiation, thus contributing to the osteoclast/osteoblast imbalance and consequently leading to OP [8]. The commonly used therapeutic agents for OP, such as denosumab and vitamin D, reduce bone resorption but also exert various adverse effects [9, 10]. Thus, there is a need to explore more effective therapeutic strategies for OP that have fewer adverse effects.

Recently, traditional Chinese medicines (TCMs) have been attracting increasing attention from researchers because of their pharmacological activities, including antiosteoporotic effects, and exhibition of fewer adverse effects. OP is thought to be mainly caused by kidney deficiency [11]; thus, kidney-tonifying TCM is expected to effectively treat OP. Yishen Bugu Ye (YSBGY) is a TCM composed of 12 types of medicinal herbs, namely, *Rhizoma Drynariae*, *Radix Polygoni Multiflori*, *Poria*, *Radix Dipsaci*, *Radix Paeoniae Alba*, *Radix Angelica sinensis*, *Radix Codonopsis*, *Radix Rehmanniae Preparata*, *Rhizoma Polygonati*, *Fructus Lycii*, *Pyritum*, and *Pericarpium Citri Reticulatae*, and has long been used in China for protecting the kidneys and improving tendon and bone strength.

The effects of these individual herbs on bone health have been reported. For example, *Rhizoma Drynariae* has been shown to inhibit the activity of tartrate-resistant acid phosphatase (TRAP) and reduce the expression of bone resorption-related genes in receptor activator of nuclear factor kappa-B ligand- (RANKL-) induced RAW264.7 cells [12]. It has been shown to increase callus formation and osseointegration, enhance bone strength at the femoral diaphysis in osteoporotic fractures, and restore the femur trabecular bone mineral density (BMD) in female Sprague-Dawley osteoporotic fracture model rats [13]. In addition, *Radix Polygoni Multiflori* has been shown to promote the proliferation and differentiation of MC3T3-E1 cells by increasing the mRNA concentrations of runt-related transcription factor-2 (Runx2), osterix (Osx), and osteopontin (OPN) and reducing those of RANKL [11]. *Radix Rehmanniae Preparata* has been reported to improve the microstructure of trabecular bone in rats with dexamethasone- (Dex-) induced OP [14]. Furthermore, the total saponins separated from *Radix Dipsaci* have been reported to induce osteoblast differentiation via Runx2 signaling [15]. However, the potential effects of YSBGY on OP and their underlying mechanisms have not yet been systematically reported.

In this study, C57BL/6 mice with Dex-induced OP were used to explore the antiosteoporotic effects of YSBGY. Notably, YSBGY was found to regulate the physiological and biochemical indexes of these mice by regulating the balance between bone resorption and formation. This finding provides experimental evidence supporting the use of YSBGY as an agent for treating OP in the clinical setting.

## 2. Materials and Methods

### 2.1. Animal Experimental Design

Ninety male C57BL/6 mice (6–8 weeks, 18–22 g) were purchased from Yis Laboratory Animal Technology Co., Ltd., Changchun, China, and kept in a standard animal house. The mice were divided into six groups (15 mice per group). The healthy control mice (*n* = 15) and YSBGY-treated healthy mice (*n* = 15) were intraperitoneally injected with 10 mL/kg of 0.9% normal saline and intragastrically administered with 10 mL/kg of 0.9% normal saline or 10 mL/kg YSBGY, respectively, every other day for seven weeks. The remaining 60 mice were intraperitoneally injected with 30 mg/kg Dex sodium phosphate every other day for seven weeks to trigger OP. The osteoporotic mice were intragastrically administered with 10 mL/kg of 0.9% normal saline (*n* = 15), 5 mL/kg of YSBGY (*n* = 15), or 10 mL/kg of YSBGY (*n* = 15) or intraperitoneally injected with 15 *μ*g/kg of estradiol (E2) (*n* = 15) every other day for seven weeks. The body weights of the mice were recorded on a weekly basis throughout the entire experimental period. The mice were euthanized shortly after the last administration, and their tibia, femur, and internal organs were immediately collected. Their liver, spleen, kidney, and thymus indexes were calculated using the following formula:

(1) $$\begin{array}{c} \text{Organ index }\left(\%\right)=\frac{\text{organ weight }\left(\text{g}\right)}{\text{body weight }\left(\text{g}\right)}. \end{array}$$

The protocol was evaluated and approved by the Animal Ethics Committee of Jilin University (SY201905007).

### 2.2. Cytokine Detection

Peripheral blood was collected from the caudal vein of each mice. The concentrations of bone gla protein (BGP; CK-E20433M), bone morphogenetic protein-2 (BMP-2; CK-E20105M), bone morphogenetic protein receptor type 2 (BMPR-2; CK-E95876M), type I collagen (COL-I; CK-E20528M), C-terminal telopeptide of type I collagen (CTX-1; CK-E20044M), OPN (CK-E20423M), tumor necrosis factor-*α* (TNF-*α*; CK-E20220M), tartrate-resistant acid phosphatase 5*β* (TRACP-5*β*; CK-E20387M), superoxide dismutase (SOD; CK-E20348), catalase (CAT; CK-E92636M), interleukin- (IL-) 1 (CK-E92636M), IL-7 (CK-E20125M), and IL-33 (CK-E93706M) in the peripheral blood were determined using the corresponding enzyme-linked immunosorbent assay kits purchased from Shanghai Yuanye Biological Technology Co., Ltd. (Shanghai, China).

### 2.3. Histological Examination of Organs and Femur Tissues

Collected organs and femur tissues of the experimental mice were fixed in 4% paraformaldehyde. The femur samples were decalcified for 3 weeks, then dehydrated and embedded in paraffin, cut into 5 *μ*m thick sections, and stained with hematoxylin-eosin (H&E) and Giemsa stain. A light microscope digital camera (Nikon Instruments, Tokyo, Japan) was used for histological examinations.

### 2.4. Microcomputed Tomography (Micro-CT) Detection

After euthanization, the femur and tibia of the experimental mice were collected and immediately fixed in formalin-saline solution. *μ*CT50 (Scanco, Switzerland) was used to evaluate the structural parameters of the trabecular and cortical regions of the femur and of the cortical region of the tibia. The parameters, including the trabecular BMD, bone volume fraction (BV/TV), trabecular thickness (Tb.Th), trabecular spacing (Tb.Sp), and trabecular number (Tb.N), were calculated by standard 3D microstructural analysis.

### 2.5. Western Blot

The collected tibias and fibulas of the experimental mice were manually pulverized in liquid nitrogen. The bone powder was homogenized in RIPA lysis buffer (Sigma-Aldrich, St. Louis, MO, USA) containing 1% protease inhibitor cocktail (Sigma-Aldrich) and 2% phenylmethanesulfonyl fluoride (Sigma-Aldrich) and centrifuged at 12000 rpm for 5 min. The supernatant was collected and subjected to protein quantification using a BCA protein kit (Merck Millipore, Billerica, MA, USA). Subsequently, 30 *μ*g of protein lysate was separated by sodium dodecyl sulfate-polyacrylamide gel electrophoresis (Sigma-Aldrich) on 10–12% gels, and the resolved proteins were transferred to methanol-activated nitrocellulose membranes (0.45 *μ*m; Bio Basic Inc., Canada). The membranes were incubated with the following primary antibodies (1 : 2000) at 4°C overnight: anti-COL-I (ab34710), anti-OPN (ab91655), anti-BMP-2 (ab6285), anti-Runx2 (12556 s), anti-TRAP5 (bs-16578R), anti-nuclear factor of activated T cell cytoplasmic 1 (NFATc1; SC-7294), and anti-*β*-actin (sc-47778) antibodies. Subsequently, the membranes were incubated with horseradish peroxidase-conjugated secondary antibodies (1 : 2000; Santa Cruz, CA, USA). The intensity of the bands was visualized using an imaging system (BioSpectrum 600, Bioss Inc., Shanghai, China). The pixel density was quantified using ImageJ software (National Institutes of Health, Bethesda, MD, USA).

### 2.6. Statistical Analysis

All data are expressed as the mean ± standard error of the mean (SEM). One-way analysis of variance (ANOVA) was performed to determine the statistically significant differences between the groups. SPSS 16.0 software (IBM Corporation, Armonk, NY, USA) was used to perform post hoc multiple comparisons (Dunn's test). *P* values of <0.05 were considered significant.

## 3. Results

### 3.1. YSBGY-Induced Protection against OP

**Figure 1:**
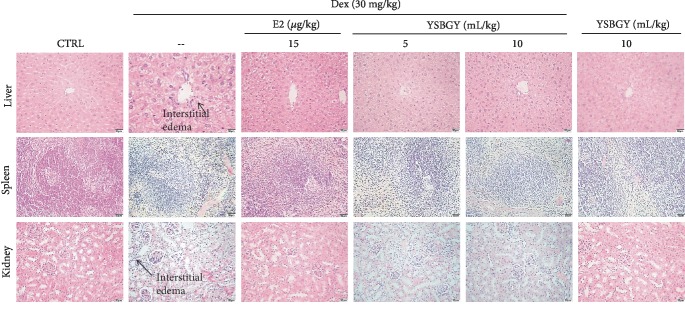
Effects of YSBGY on the structures of the liver, spleen, and kidney of OP mice visualized by H&E staining (*n* = 6; 200x, scale bar: 50 *μ*m).

Low body weights were observed in mice with Dex-induced OP (hereafter referred to as OP mice) (*P* < 0.001, Table 1), which were not reversed after YSBGY or E2 administration (Table 1). Liver swelling and spleen and thymus shrinking were also noted in OP mice (*P* < 0.001, Table 1). Comparatively, YSBGY reduced the high liver index and attenuated the decrease in the spleen index in OP mice (*P* < 0.05, Table 1) but did not affect the kidney and thymus indexes (Table 1). E2 showed no effects on these organ indexes in OP mice (Table 1). Furthermore, interstitial edema was observed in the liver and kidneys of OP mice, which was significantly reduced by YSBGY (Figures 1(a) and 1(c)). No significant differences were observed in the spleen indexes between the groups (Figure 1(b)). YSBGY (10 mL/kg) administration in healthy mice showed no significant effects on body weights, organ indexes, or bone structures (Table 1 and Figure 1).

**Figure 2:**
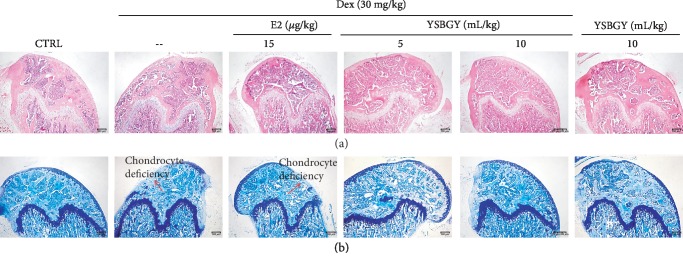
Effects of YSBGY on the histological changes in the femur of OP mice visualized by (a) H&E staining and (b) Giemsa staining (*n* = 6; 40x, scale bar: 200 *μ*m).

H&E staining revealed that the number of trabecular bone elements in the femur of OP mice was significantly reduced, and this reduction was reversed by seven-week YSBGY and E2 treatments (Figure 2(a)). Giemsa staining revealed that seven-week YSBGY administration enhanced the number of chondrocytes in the femur of OP mice (Figure 2(b)), whereas E2 had no significant effect on this parameter (Figure 2(b)). YSBGY administration in healthy mice showed no significant effects on these bone parameters (Figure 2).

**Figure 3:**
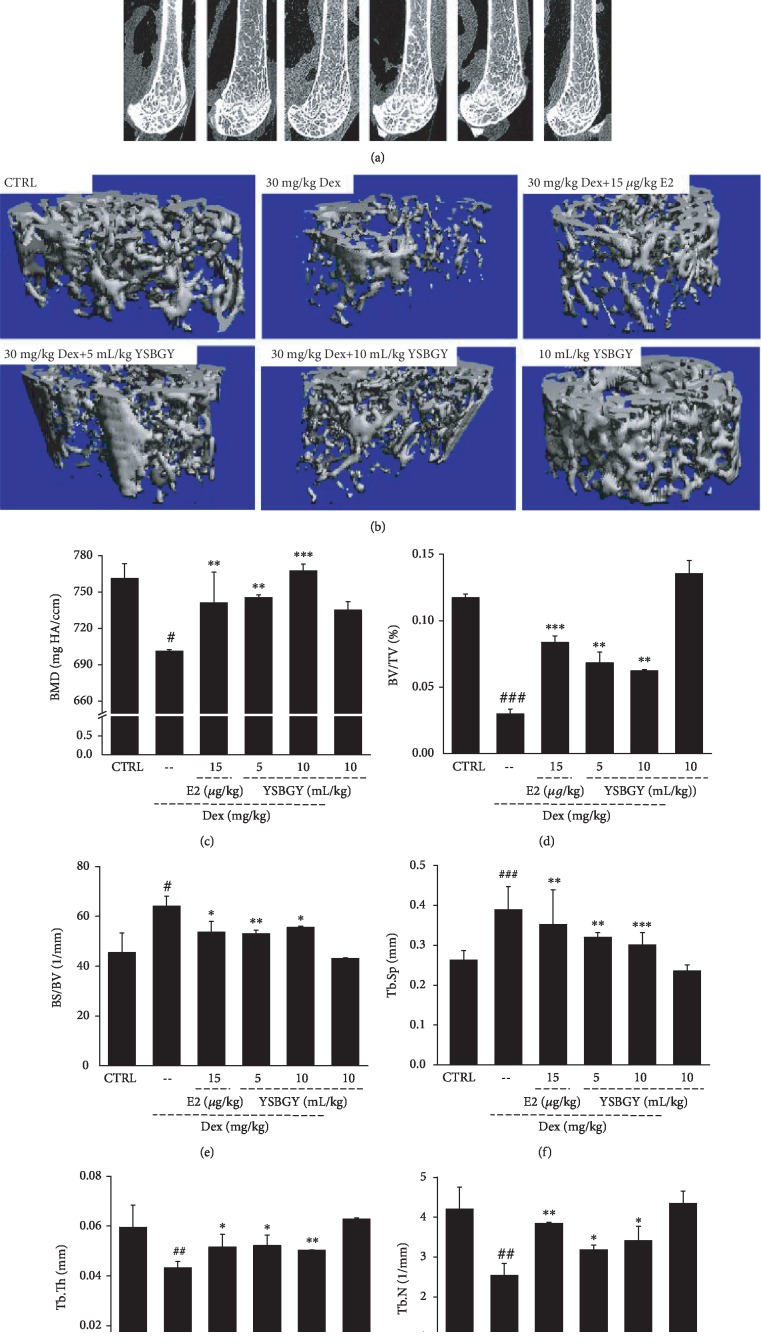
YSBGY-induced protection against OP revealed by the bone morphological changes and osteoporotic indexes of the femur in OP mice. (a) Micro-CT images of the femur in OP mice. (b) 3D reconstructed images of the trabecular bone in the femur of OP mice. The (c) BMD, (d) BV/TV, (e) BS/BV, (f) Tb.Sp, (g) Tb.Th, and (h) Tb.N of the femur in all groups. Data are expressed as means ± SEM (*n* = 6) and analyzed using a one-way ANOVA. ^#^*P* < 0.05, ^##^*P* < 0.01, and ^###^*P* < 0.001*vs.* control mice; ^∗^*P* < 0.05, ^∗∗^*P* < 0.01, and ^∗∗∗^*P* < 0.001*vs.* OP mice.

**Figure 4:**
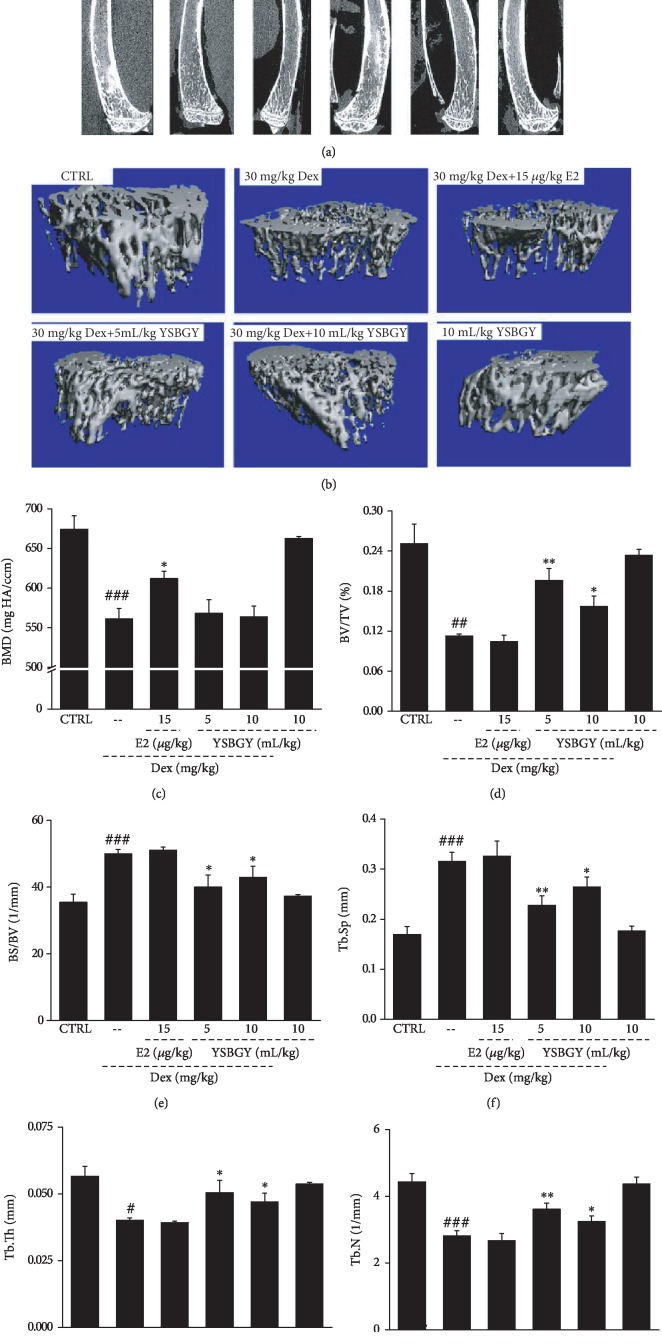
YSBGY-induced protection against OP revealed by the bone morphological changes and the osteoporotic indexes of the tibia in OP mice. (a) Micro-CT images of the tibia in OP mice. (b) 3D reconstructed images of the trabecular bone in the tibia of OP mice. The (c) BMD, (d) BV/TV, (e) BS/BV, (f) Tb.Sp, (g) Tb.Th, and (h) Tb.N of the tibia in all groups. Data are expressed as means ± SEM (*n* = 6) and analyzed using a one-way ANOVA. ^#^*P* < 0.05, ^##^*P* < 0.01, and ^###^*P* < 0.001*vs.* control mice; ^∗^*P* < 0.05, ^∗∗^*P* < 0.01, and ^∗∗∗^*P* < 0.001*vs.* OP mice.

Next, the distal trabecular and cortical bone elements of the femurs and tibias were evaluated by micro-CT analysis. The femurs and tibias of OP mice had thinner cortical bone and sparser trabecular bone compared with those in heathy control mice (Figures 3(a), 3(b), 4(a), and 4(b)). YSBGY promoted the cortical bone thickness and trabecular bone density in the femurs and tibias of OP mice (Figures 3(a), 3(b), 4(a), and 4(b)). Furthermore, seven-week YSBGY administration enhanced the BV/TV (*P* < 0.05, Figures 3(d) and 4(d)), Tb.Th (*P* < 0.05, Figures 3(g) and 4(g)), and Tb.N (*P* < 0.05, Figures 3(h) and 4(h)) and reduced the BS/BV (*P* < 0.05; Figures 3(e) and 4(e)) and Tb.Sp (*P* < 0.05, Figures 3(f) and 4(f)) in the femurs and tibias of OP mice. YSBGY also attenuated the loss of BMD in the femur (*P* < 0.01; Figure 3(c)), but not in the tibia (Figure 3(d)), of OP mice. E2 significantly improved the BV/TV (*P* < 0.001, Figure 3(d)), Tb.Th (*P* < 0.05, Figure 3(g)), and Tb.N (*P* < 0.01, Figure 3(h)) and reduced the BS/SV (*P* < 0.05, Figure 3(e)) and Tb.Sp (*P* < 0.01, Figure 3(f)) in the femur of OP mice but did not significantly affect these parameters in their tibia (Figures 4(d)–4(h)). E2 significantly enhanced the BMD in both the femur (*P* < 0.01, Figure 3(c)) and the tibia (*P* < 0.05, Figure 4(c)) of OP mice. YSBGY administration in healthy mice showed no effects on these bone parameters (Figures 3 and 4).

### 3.2. YSBGY-Induced Protection against OP via the Regulation of the Balance between Bone Resorption and Formation

**Figure 5:**
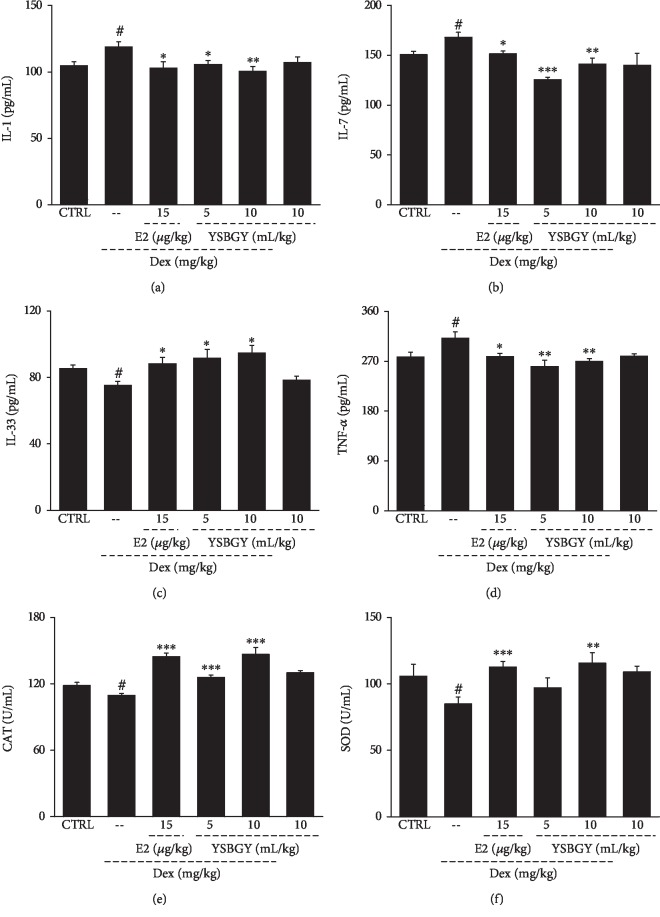
Regulation of the serum levels of inflammatory cytokines in OP mice by YSBGY. YSBGY reduced the serum levels of (a) IL-1, (b) IL-7, and (d) TNF-*α* and increased those of (c) IL-33, (e) CAT, and (f) SOD in OP mice. Data are expressed as means ± SEM (*n* = 10) and analyzed using a one-way ANOVA. ^#^*P* < 0.05*vs.* control mice; ^∗^*P* < 0.05, ^∗∗^*P* < 0.01, and ^∗∗∗^*P* < 0.001*vs.* OP mice.

Compared with the healthy control mice, the OP mice showed higher serum concentrations of IL-1, IL-7, and TNF-*α* and lower serum concentrations of IL-33 (*P* < 0.05, Figures 5(a)–5(d)). YSBGY administration resulted in 11.3%, 15.9%, and 16.5% decrease in the serum concentrations of IL-1, IL-7, and TNF-*α* and a 21.9% increase in those of IL-33 in OP mice (*P* < 0.05, Figures 5(a)–5(d)). Furthermore, YSBGY significantly increased the serum concentrations of CAT (*P* < 0.001, Figure 5(e)) and SOD (*P* < 0.01, Figure 5(f)) in OP mice.

**Figure 6:**
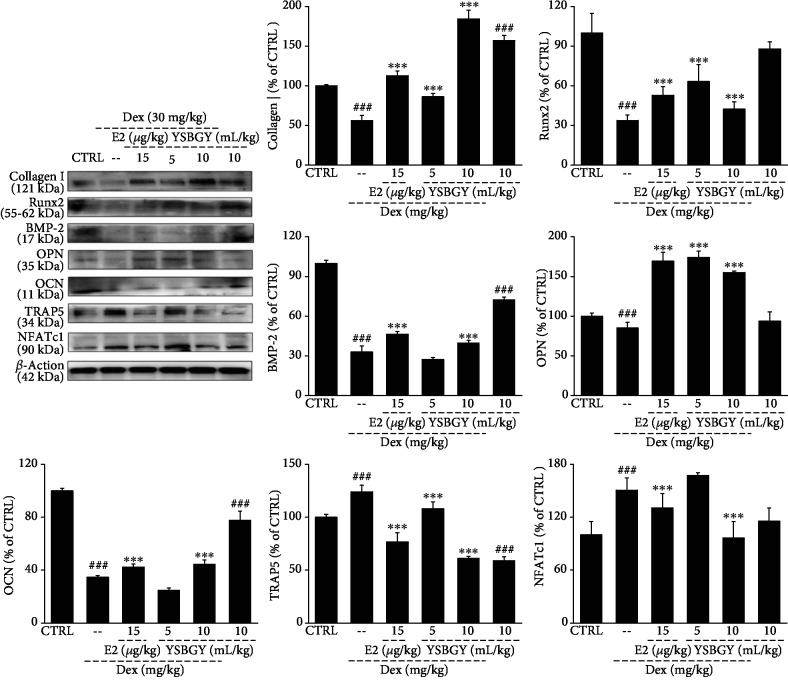
Regulation of the concentrations of osteoblast and osteoclast differentiation-related proteins in the femur and tibia tissues of OP mice by YSBGY. YSBGY increased the concentrations of osteoblast differentiation-related proteins, including COL-I, Runx2, OCN, OPN, and BMP-2, and decreased those of osteoclast differentiation-proteins, including TRAP5 and NFATc1, in the femur and tibia tissues of OP mice. The protein concentrations were normalized to *β*-actin and total protein concentrations. Data are expressed as means ± SEM (*n* = 6) and analyzed using a one-way ANOVA. ^###^*P* < 0.001*vs.* control mice; ^∗∗∗^*P* < 0.001*vs.* OP mice.

We also found that seven-week YSBGY administration resulted in a 22.0%, 13.6%, 26.5%, 12.9%, 20.2%, and 43.2% decrease in the serum concentrations of COL-I, BGP, OCN, OPN, BMP-2, BMPR-2, and CTX-1 and a 16.5% increase in those of TRACP-5*β* (*P* < 0.05, Table 2). Furthermore, the tibias and femurs of OP mice exhibited decreased concentrations of COL-I, Runx2, BMP-2, OPN, and OCN and increased concentrations of TRAP5 and NFATc1 (*P* < 0.001, Figure 6); however, these changes were significantly reversed by seven-week YSBGY or E2 administration (*P* < 0.001, Figure 6). Compared with the healthy control mice, the YSBGY-treated healthy mice showed increased concentrations of COL-I and reduced levels of OCN and TRAP5 in their bone tissues (*P* < 0.001, Figure 6).

## 4. Discussion

OP is a metabolic bone disorder that increases the risk of fracture [1]. Long-term and high-dose administration of Dex has been reported to cause osteoblast apoptosis, osteopenia, and osteonecrosis and promote osteoclast proliferation, leading to OP [16, 17]. Therefore, Dex was used to induce OP in C57BL/6 mice in this study to investigate the effects of the TCM YSBGY on OP.

Micro-CT findings showed that YSBGY prevented Dex-induced OP in mice, as evidenced by its positive effects on the femur and tibia structures and related parameters. BMD is an important bone parameter commonly used in the clinical evaluation of OP [18]. BV/TV and BS/BV are commonly used indicators of cortical and trabecular bone mass that can directly or indirectly reflect the bone content [19]. The trabecular bone is an extension of the cortical bone and has an irregular three-dimensional network structure in the bone marrow cavity that supports the hematopoietic tissue. Tb.N, Tb.Th, and Tb.Sp are the main indicators used for evaluating the spatial morphology of trabecular bone [19]. In OP, bone catabolism is greater than bone anabolism, leading to decreased Tb.N and Tb.Th and increased Tb.Sp [20]. Consistent with previous findings, our results showed that the BV/TV, Tb.Th, and Tb.N were suppressed, whereas the BS/BV and Tb.Sp were significantly enhanced in the femurs and tibias of mice with Dex-induced OP, and all these changes were reversed by the seven-week YSBGY treatment.

BMP implantation in the muscles has shown to induce ectopic bone formation and stimulate osteoblast differentiation in various cell types [21]. Increased BMP concentrations have been reported to increase bone formation in osteoporotic mice, whereas decreased BMP concentrations tend to reduce the bone mass [22, 23]. Mice with BMP-2 deficiency have shown inadequate bone formation, but short-term administration of BMP-2 can reverse the bone loss in osteoporotic mice [22]. As a major transcription factor, Runx2 regulates embryonic skeletal development and postnatal osteoblast function. Our results suggest that YSBGY increased the differentiation of preosteoblasts into mature osteoblasts and promoted bone formation by increasing the expression of Runx2 and BMP-2. In addition, YSBGY increased the concentrations of other important markers such as COL-I (a type of collagen) and OPN and OCN (two noncollagen proteins), thus promoting the differentiation of preosteoblasts. During osteoclast formation, RANKL stimulation leads to NFATc1 induction, which regulates osteoclast differentiation [24]. TRAP (an enzyme abundantly expressed in OP), inflammatory macrophages, and dendritic cells are major markers of osteoclast formation [25]. In our study, YSBGY treatment was found to significantly inhibit the expression of NFATc1 and TRAP5 in OP mice.

In chronic inflammation, the activation of immune cells can cause the overexpression of bone resorption cytokines, which in turn stimulate the formation and activation of osteoclasts. IL-1, mainly produced by monocytes, is involved in multiple steps of bone metabolism such as differentiation, multinucleation, activation, and survival of osteoclasts [26] and is implicated in various bone loss-related related diseases including OP, cancer-induced osteolysis, and rheumatoid arthritis [27]. IL-1 stimulates osteoblasts to produce RANKL, a key promotor of osteoclastogenesis. TNF-*α* promotes stromal cell expression in osteoblasts and stimulates osteoclast activation [28]. Osteoclasts are bone-specific aggregated multinuclear macrophages derived from monocytes when stimulated by cytokines such as RANKL and TNF-*α* [29]. IL-7 primarily acts on macrophages, lymphoid B cells, and lymphoid T cells and plays an essential role in cell survival, proliferation, and differentiation [30]. IL-7/IL-7 receptor enhances bone resorption by activating T cells and T cell-dependent osteoclastogenesis [31]. IL-33 is a member of the IL-1 family and functions as an important bone-protecting cytokine. IL-33/ST2 signaling reduces bone loss, inhibits osteoclast formation, promotes osteoblast function, interferes with the production of RANKL and macrophage colony-stimulating factor, and decreases NFATc1 expression in osteoclast precursors [32, 33]. In this study, the serum concentrations of IL-1, IL-7, and TNF-*α* were increased and those of IL-33 were decreased in mice with Dex-induced OP compared with healthy control mice, and all of these changes were reversed to the normal state by the seven-week YSBGY treatment.

Our study has a limitation. We could not determine the signaling pathways that control the changes in inflammation- and osteoblast and osteoclast differentiation-related factors caused by YSBGY, a complex TCM, because investigation of the underlying mechanism is difficult in *in vitro* experiments.

In summary, we have confirmed the antiosteoporotic effects of YSBGY in C57BL/6 mice with Dex-induced OP. Our findings suggest that these effects could be related to YSBGY-induced modulation of the osteoblast/osteoclast balance and inflammatory factor levels. These findings provide experimental evidence supporting the use of YSBGY as the main agent to treat bone formation-related diseases in the clinical setting.

## Figures and Tables

**Table 1:** Effects of YSBGY on the body weight and organ indexes of OP mice.

	Days	CTRL	Dex (30 mg/kg)				YSBGY (10 mL/kg)
			—	E2 (15 *μ*g/kg)	YSBGY (mL/kg)		
					5	10	
Body weight (g)	0 day	22.3 ± 0.3	22.7 ± 0.2	22.5 ± 0.3	22.2 ± 0.3	22.6 ± 0.2	22.8 ± 0.3
	7^th^ day	23.2 ± 0.3	22.1 ± 0.1^###^	22.0 ± 0.2	21.9 ± 0.2	21.8 ± 0.2	22.7 ± 0.3
	14^th^ day	23.5 ± 0.4	21.9 ± 0.3^###^	21.9 ± 0.2	21.3 ± 0.2	21.7 ± 0.2	23.7 ± 0.4
	21^st^ day	24.2 ± 0.3	22.2 ± 0.2^###^	22.4 ± 0.3	22.1 ± 0.2	22.2 ± 0.2	24.4 ± 0.4
	28^th^ day	24.5 ± 0.3	21.8 ± 0.2^###^	22.0 ± 0.3	21.6 ± 0.3	21.7 ± 0.2	24.7 ± 0.4
	35^th^ day	25.1 ± 0.3	21.3 ± 0.4^###^	21.1 ± 0.2	20.8 ± 0.4	21.4 ± 0.2	24.9 ± 0.4
	42^nd^ day	25.8 ± 0.3	21.8 ± 0.4^###^	21.6 ± 0.2	21.2 ± 0.2	21.4 ± 0.2	25.4 ± 0.5
	49^th^ day	26.6 ± 0.4	21.9 ± 0.2^###^	21.9 ± 0.2	21.6 ± 0.2	21.4 ± 0.2	25.7 ± 0.5

Organ index (%)	Liver	4.88 ± 0.15	7.87 ± 0.14^###^	7.50 ± 0.08	7.44 ± 0.11	7.32 ± 0.12^∗^	4.78 ± 0.19
	Spleen	0.35 ± 0.01	0.27 ± 0.01^###^	0.28 ± 0.03	0.26 ± 0.01	0.39 ± 0.03^∗^	0.31 ± 0.01
	Kidney	1.59 ± 0.06	1.57 ± 0.02	1.59 ± 0.02	1.53 ± 0.03	1.57 ± 0.03	1.49 ± 0.06
	Thymus	0.221 ± 0.017	0.066 ± 0.009^###^	0.077 ± 0.007	0.061 ± 0.005	0.056 ± 0.006	0.201 ± 0.014

The data were analyzed using a one-way ANOVA and expressed as means ± SEM (*n* = 15). ^###^*P* < 0.001*vs.* control mice; ^∗^*P* < 0.05*vs.* OP mice.

**Table 2:** Effects of YSBGY on osteoblast and osteoclast differentiation-related factors in the peripheral blood of OP mice.

Groups	CTRL	Dex (30 mg/kg)				YSBGY (10 mL/kg)
		—	E2 (15 *μ*g/kg)	YSBGY (mL/kg)		
				5	10	
Collagen I (ng/mL)	14.0 ± 0.7	10.9 ± 0.7^##^	13.5 ± 0.8^∗^	12.1 ± 0.8	13.3 ± 0.8^∗^	12.7 ± 0.7
BGP OCN (ng/mL)	4.30 ± 0.16	3.82 ± 0.06^#^	4.46±0.14^∗∗^	4.34±0.09^∗∗^	4.62±0.14^∗∗^	4.69 ± 0.10
OPN (ng/mL)	109.2 ± 3.4	94.7 ± 5.6^#^	118.4±5.4^∗∗^	119.8 ± 7.3^∗^	121.0±4.0^∗∗^	122.5 ± 5.0
BMP-2 (ng/mL)	9.60 ± 0.19	8.76 ± 0.21^#^	9.84 ± 0.33^∗^	9.89 ± 0.31^∗^	10.41±0.34^∗∗^	11.38 ± 1.50
BMPR-2 (ng/mL)	3.83 ± 0.12	3.41 ± 0.12^#^	4.21 ± 0.29^∗^	4.10±0.17^∗∗^	4.49±0.13^∗∗∗^	4.42 ± 0.59
TRACP-5*β* (U/L)	3.85 ± 0.15	4.61 ± 0.26^#^	3.86 ± 0.10^∗^	3.54±0.16^∗∗^	3.85 ± 0.18^∗^	3.63 ± 0.08
CTX-1 (ng/mL)	4.73 ± 0.24	3.84 ± 0.22^#^	4.65 ± 0.29^∗^	6.31±0.15^∗∗∗^	5.50±0.30^∗∗∗^	4.74 ± 0.27

The data were analyzed using a one-way ANOVA and expressed as means ± SEM (*n* = 10). ^#^*P* < 0.05 and ^##^*P* < 0.01*vs.* control mice; ^∗^*P* < 0.05, ^∗∗^*P* < 0.01, and ^∗∗∗^*P* < 0.001*vs.* OP mice.

## Data Availability

The data that support the findings of this study are available from the corresponding author upon reasonable request. The source data underlying Tables 1 and 2 and Figures 1–6 are provided as a Source Data file.
