# Corrigendum to “The Antiosteoporosis Effects of Yishen Bugu Ye Based on Its Regulation on the Differentiation of Osteoblast and Osteoclast”

**DOI:** 10.1155/2021/9824165

**Published:** 2021-04-12

**Authors:** Yangyang Li, Yongfeng Zhang, Weiqi Meng, Yutong Li, Tao Huang, Di Wang, Min Hu

**Affiliations:** ^1^Department of Orthodontics, School and Hospital of Stomatology, Jilin University, Changchun 130021, China; ^2^School of Life Sciences, Jilin University, Changchun 130012, China; ^3^Changchun University of Chinese Medicine, Changchun 130117, China

In the article titled “The Antiosteoporosis Effects of Yishen Bugu Ye Based on Its Regulation on the Differentiation of Osteoblast and Osteoclast” [1], the authors identified a mistake in Figure 1, where panel 2 of the liver samples was duplicated with panel 6. The authors confirm that this does not affect the results and conclusions of the article, and the corrected Figure 1 is as follows:

## Figures and Tables

**Figure 1:**
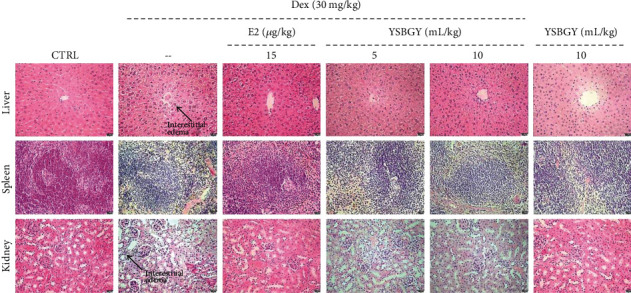
Effects of YSBGY on the structures of the liver, spleen, and kidney of OP mice visualized by H&E staining (*n* = 6; 200x, scale bar: 50 *μ*m).
